# Extensive Multiorgan and Neurological Involvement on F-18 FDG PET-CT in a Case of Rosai-Dorfman Disease

**DOI:** 10.1055/s-0044-1787962

**Published:** 2024-07-15

**Authors:** Sireesha Polisetty, Ramyapriya R., Hema latha D.S, Tekchand Kalawat

**Affiliations:** 1 Department of Nuclear Medicine, Sri Venkateswara Institute of Medical Sciences, Tirupati, Andhra Pradesh, India

**Keywords:** benign proliferative disorders, F18-FDG PET CT, molecular imaging, multisystem involvement, Rosai-Dorfman disease

## Abstract

Rosai-Dorfman disease (RDD) is a rare benign proliferative disorder. Lymph nodes are the usual and common sites of involvement. Involvement of the extranodal site is also documented in the literature. 18F-fluorodeoxyglucose positron emission tomography computed tomography (F18-FDG PET CT) is a valuable whole-body imaging modality in staging and treatment response of various lymphoproliferative and solid organ malignancy. Similarly, PET CT survey can detect the involved sites of various body systems, infective or inflammatory diseases, and provide guidance for biopsy and to reach to diagnosis. Here, we present a case of RDD, who presented with neurological manifestations and on F18-FDG PET CT, diagnosed with multiorgan involvement.

## Introduction

Rosai-Dorfman disease (RDD) is a rare benign disorder, first described by Juan Rosai and Ronald Dorfman in 1969. It is also known as sinus histiocytosis with massive lymphadenopathy and predominantly seen in young males (median age of 20.4 years). 1_._

Most common clinical presentation is a massive bilateral and painless cervical lymphadenopathy (57%) with fever, night sweats, and weight loss. Extrnnodal disease involvement is seen in 43% of cases. 2

RDD often presents similarly to other diseases and malignancies like histiocytosis, immunoglobulin G (IgG)-related disease, and lymphoma, which can lead to misdiagnosis easily, making it a diagnosis of exclusion. 3

Histopathological examination (HPE) and immunohistochemistry (IHC) are considered as the gold standard for diagnosis. These methods are able to differentiate RDD from other infectious and lymphoproliferative disorders by CD68 and S-100 positivity. 4 5

Radiological conventional imaging modalities like plain X-ray film, ultrasonography, computerized tomography (CT), and magnetic resonance imaging (MRI) are nonspecific and can be variable, due to wide disease spectrum. 6 However, 18F-fluorodeoxyglucose positron emission tomography CT (F18-FDG PET CT), being a whole-body screening tool, is valuable for detecting the involved sites, guidance for biopsy, and further assessment of treatment response. 7

## Case Report

A 50-year-old male patient with history of hypertension has been experiencing hallucinations for 1 year. Initially, he had difficulty in identifying faces but was able to identify the voice. These symptoms were intermittent and accompanied by complaints of palpitations and suicidal tendencies.

**Fig. 1:**
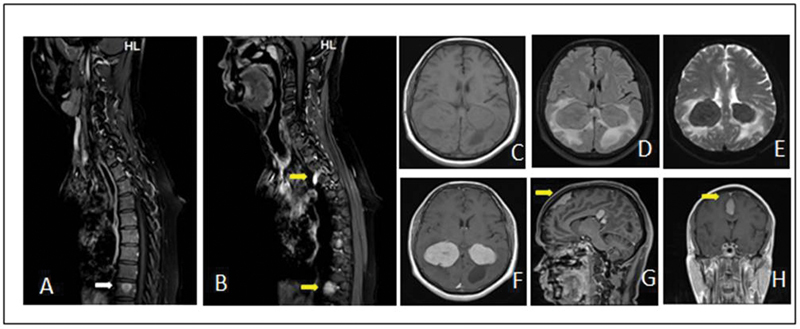
Magnetic resonance imaging (MRI) spine and brain plain and contrast study: ( **A** , **B** ) images show enhancing lesion in vertebral body (white arrow) and paravertebral region on right side with extension into neural foramina (yellow arrow) at T3/T4 and T9/T10 levels. ( **C** – **F** ) Well-defined iso- to hypointense lesion measuring 4.6 × 4.0 × 7.1 cm on T1 and T2 with no restricted diffusion on diffusion-weighted imaging (DWI). The lesion shows homogenous enhancement on postcontrast study. ( **G** , **H** ) Dural-based extra-axial lesion (yellow arrow) along the falx measuring 4.8 × 1.1 cm in the frontal midline region.

On clinical examination his Glasgow Coma Scale was normal, pupils dilated normally, and limbs power was normal. Later, evaluation with MRI brain showed enhancing lesion in bilateral and lateral ventricles, and dural-based lesion along the falx with enhancing lesion in neural foramina at the T3/T4 and T9/T10 levels ( Fig. 1 ).

**Fig. 2:**
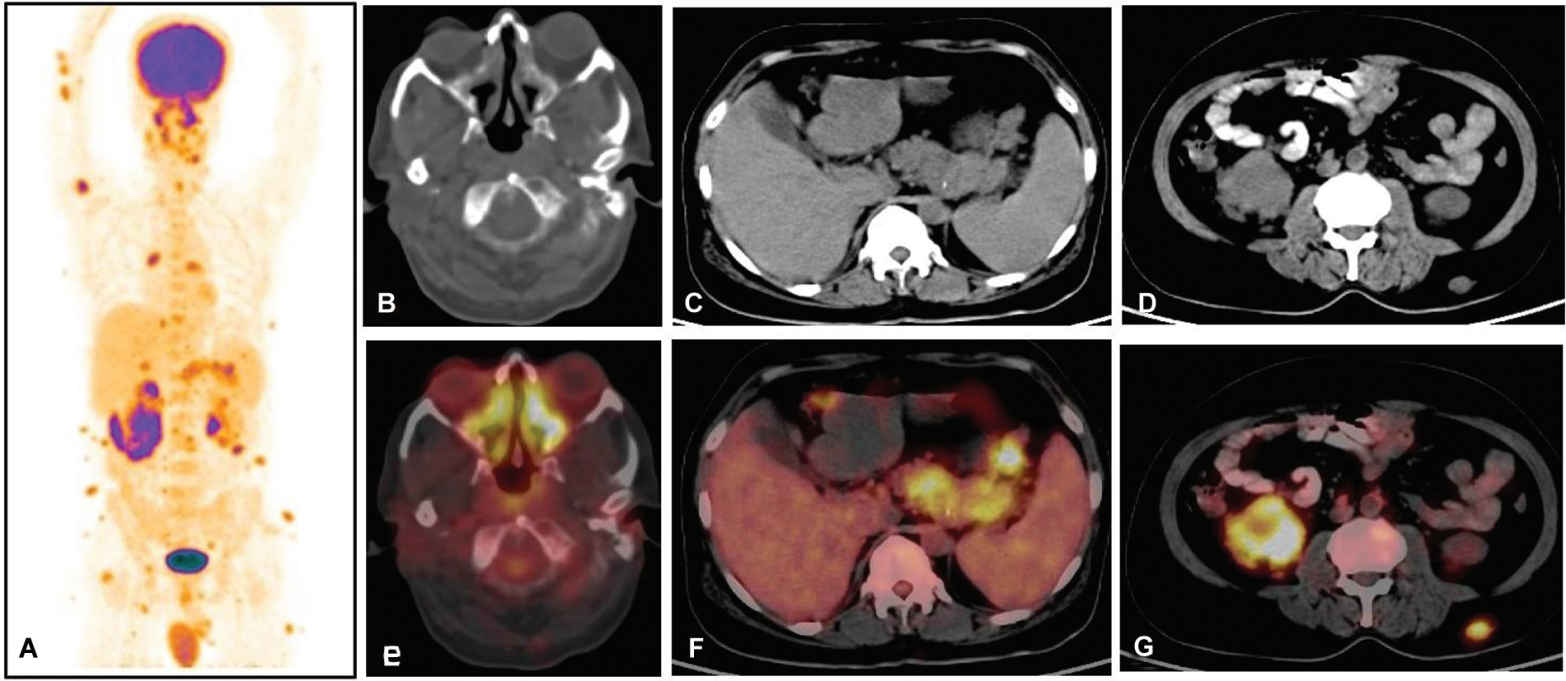
18F-fluorodeoxyglucose (F18-FDG) whole body positron emission tomography computed tomography (PET CT) maximum intensity projection image ( **A** ) shows multiple FDG-avid subcutaneous lesions in body and diffuse increased uptake in enlarged right testis. CT image ( **B** – **D** ) and PET CT image ( **E** – **G** ) show diffuse increased FDG concentration in bilateral paranasal mucosal thickening in the body and tail region of pancreas and right kidney parenchyma, respectively.

He was provisionally diagnosed with Erdheim-Chester disease and subsequently underwent F18-FDG PET CT. The scan showed metabolically active lymphadenopathy both above and below the diaphragm, soft tissue thickening of paranasal sinuses, hyperdense lesions in the brain, multiple deposits in the pleura, pancreas, kidney, and testes, and extensive subcutaneous deposits with skeletal and vascular involvement ( Fig. 2 ).

**Fig. 3:**
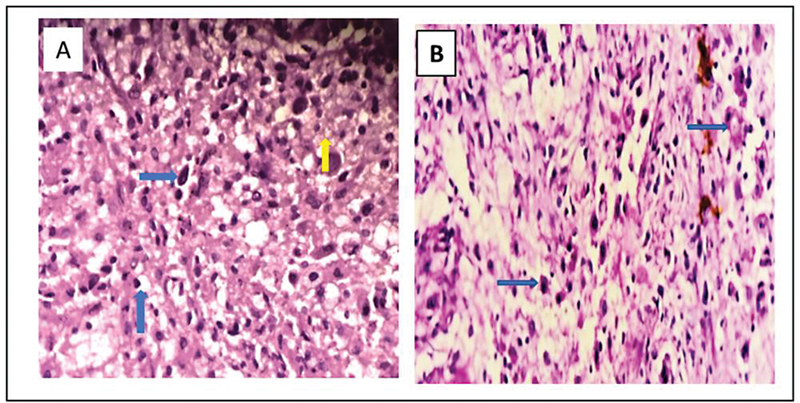
Hematoxylin and eosin (H&E) 400× image ( **A** ) showing dense infiltrate of histiocytes with abundant foamy cytoplasm (blue arrows) admixed with multinucleated giant cells (yellow arrow). ( **B** ) Dense infiltrate of histiocytes admixed lymphocytes and plasma cells (blue arrows).

HPE from the left thigh subcutaneous deposit revealed a lesion composed of sheets of foamy histiocytes arranged in lobules, few foci showed lymphoplasmacytic infiltrate and multinucleate giant cells. Later, an IHC test confirmed S-100 and CD68 positivity and a diagnosis of RDD was made ( Fig. 3 ).

## Discussion

RDD is a rare non-Langerhans cell histiocytosis characterized by the accumulation of activated histiocytes within affected tissues with a prevalence of 1:200,000. It is more common among individuals of African descent. 8 The clinical course of RDD is unpredictable, marked by episodes of exacerbation and remission. 9

Molecular studies in RDD showed recurrent mutations involving KRAS and MAP2K1 in one-third cases and this was highlighted in a retrospective study by Garces et al in 2017. 10 Genomic analysis was not performed in our case; however, literature suggests these mutations could explain the extensive disease presentation.

Most common site of extranodal involvement is the skin (10%) followed by paranasal sinuses and bones (5–10%), intracranial involvement (< 5%), intrathoracic involvement (2%), and very rarely gastrointestinal involvement (< 1%). 11 12 13

MRI of the brain shows contrast enhancement with variable signal intensities in T1 and T2 images. In our case, MRI showed enhancing lesions in lateral ventricles and dural-based lesion along the falx with enhancing lesions in paranasal and neural foramina at the T3/T4 and T9/T10 levels. Raslan et al, in a case series, reported central nervous system involvement in over 50 RDD patients with intracranial involvement being more common than spinal. 14

True disease burden assessment is not always possible on conventional imaging, due to limited field of view. As a result, the best treatment options might not be possible if extranodal sites of involvement are missed. 15 Nuclear medicine imaging modalities like gallium 67 (Ga67) scan and F18-FDG PET CT showed potential role in demonstrating the extent of disease and choosing the best treatment. 16

F18-FDG PET CT in RDD commonly shows intense metabolic activity due to high glucose metabolism of proliferating histiocytes and other inflammatory cells. Fathala et al in 2021 highlighted the role of F18-FDG PET CT as a single-stop imaging tool to assess the disease burden. 17 In our case report, prior to PET CT only clinically palpable cervical lymph nodes, subcutaneous nodules, brain, spinal cord, and vertebral involvement were known. However, PET CT documented extensive lymphadenopathies both above and below the diaphragm as well as other sites like the pleura, pancreas, kidney, testes, and vascular structures.

In RDD arterial and venous structures typically remain unaffected. Rare presentations of vascular involvement at mediastinal blood vessels and femoral artery were reported by Baldi et al, respectively. 18 In our case, we document abdominal aorta involvement, which is a very rare site documented so far.

In RDD bone involvement is rare (10%) and in 2020, Fard-Esfahani et al observed lytic bone lesions as the common presentation. 19 However, our case is unique, showing multiple sclerotic skeletal lesions, a rare presentation.

Mahajan et al reviewed 109 F18-FDG PET CT scans in 27 RDD, and observed additional skeletal and pleural involvement missed on conventional imaging in 6 patients,, resulting change in management of 41% of cases. F18-FDG PET CT has a potential role in initial assessment, disease extent evaluation, and treatment response monitoring. 20 Our case report also highlights its role in guiding biopsy site thereby improving diagnostic sensitivity.

RDD usually follows a benign and self-limiting course. Treatment is indicated only when nodal disease causing life-threatening complications or extranodal disease involving vital organs. 21 Surgery options may be warranted for symptomatic control. Systemic corticosteroids are usually helpful in decreasing nodal size and symptoms. 22 Drugs that specifically target cytokines (tumor necrosis factor-αand interleukin-6), such as cladribine, have been found to be effective in recurrent, refractory, or severe cases of RDD. 23 24

## Conclusion

RDD is a rare benign disease, with multiorgan involvement being even rarer. Often, this disease could be easily confused with other lymphoproliferative disorders like histiocytosis, IgG-related disease, and lymphoma, and require thorough evaluation. Whole body F18-FDG PET CT is a valuable imaging modality in staging, precisely identifying affected sites, guiding biopsy, and monitoring treatment response.
